# Mathematic Modeling for Optimum Conditions on Aflatoxin B_1_ Degradation by the Aerobic Bacterium *Rhodococcus erythropolis*

**DOI:** 10.3390/toxins4111181

**Published:** 2012-11-06

**Authors:** Qing Kong, Cuiping Zhai, Bin Guan, Chunjuan Li, Shihua Shan, Jiujiang Yu

**Affiliations:** 1 School of Food Science and Engineering, Ocean University of China, Qingdao, Shandong 266003, China; Email: tomato-yy@163.com (C.Z.); guanbin@ouc.edu.cn (B.G.); 2 Shandong Peanut Research Institute, Qingdao, Shandong 266100, China; Email: peanutlab@163.com (C.L.); shhshan@sina.com (S.S.); 3 US Department of Agriculture (USDA), Agricultural Research Service (ARS), Southern Regional Research Center, New Orleans, LA 70124, USA; Email: Jiujiang.yu@ars.usda.gov

**Keywords:** *Rhodococcus erythropolis*, degradation efficiency, optimization, Plackett–Burman design, central composite design, response surface methodology, aflatoxins

## Abstract

Response surface methodology was employed to optimize the degradation conditions of AFB_1_ by *Rhodococcus erythropolis* in liquid culture. The most important factors that influence the degradation, as identified by a two-level Plackett-Burman design with six variables, were temperature, pH, liquid volume, inoculum size, agitation speed and incubation time. Central composite design (CCD) and response surface analysis were used to further investigate the interactions between these variables and to optimize the degradation efficiency of *R. erythropolis* based on a second-order model. The results demonstrated that the optimal parameters were: temperature, 23.2 °C; pH, 7.17; liquid volume, 24.6 mL in 100-mL flask; inoculum size, 10%; agitation speed, 180 rpm; and incubation time, 81.9 h. Under these conditions, the degradation efficiency of *R. erythropolis* could reach 95.8% in liquid culture, which was increased by about three times as compared to non-optimized conditions. The result by mathematic modeling has great potential for aflatoxin removal in industrial fermentation such as in food processing and ethanol production.

## 1. Introduction

Mycotoxins are secondary metabolites produced by *Aspergillus*, *Fusarium* and other fungal species that may be present in agricultural commodities and other food [1]. Aflatoxins (AFs) are produced mainly by *Aspergillus flavus* and *A. parasiticus*. There are mainly four types of aflatoxins, namely AFB_1_, AFB_2_, AFG_1_, and AFG_2_. They are detected in most food, such as corn, peanuts, tree nuts, milk and seafood [2,3]. Among the aflatoxins, aflatoxin B_1_ (AFB_1_) is the most widespread and so the most investigated mycotoxin because of its hepatotoxic, hepatocarcinogenic, mutagenic and teratogenic properties [4,5]. Due to the great health risk posed by aflatoxin contamination of food and feed, many countries have established Maximum Residue Limits (MRL) allowed in food and feed supplies for consumption and trading. The European Union has set more stringent regulations that limits the amount of aflatoxin B_1_ to no more than 5 ng g^−1^ in nuts and oils and zero tolerance of total aﬂatoxins in infant formula [6]. The U.S. Food and Drug Administration has set the amount of total aﬂatoxins for interstate trading and consumption to no more than 20 ng g^−1^. To minimize potential human exposure to aflatoxins, numerous strategies have been used to control the growth of fungi and inhibit aflatoxin formation [7]. Physical methods such as adsorption, heating, UV light, and ionizing radiation are effective only to some degree. Chemical degradation by the addition of chlorinating, oxidizing or hydrolytic agents, are not widely accepted, except ammoniation, which requires expensive equipments and may result in loss of nutritional quality and flavor [8]. Therefore, it is important to find a practical, cost effective and non-toxic method for aflatoxin removal. Use of natural plant extracts and biological methods are one kind of efficient, low-hazardous method for removing toxins [9]. Natural products of plants, such as the extracts of *Agave* species [10], *Garcinia indica* extract [11] and *Satureja hortensis* L. essential oil [12] can inhibit the growth of *Aspergillus flavus* and aflatoxin biosynthesis. Bacteria isolated from almonds also showed inhibition of *A. flavus* growth [13]. Previous studies have demonstrated that some bacterial species could degrade aflatoxin. *Flavobacterium* NRRL B-184 could detoxify aflatoxin in contaminated materials irreversibly [14]. A strain of *Mycobacterium fluoranthenivorans* screened from soil of a former coal gas plant was able to degrade aflatoxin B_1_ [15]. Several species of Actinomycetales could degrade aflatoxins, too. Recently, two families of F_420_ H_2_-dependent reductases from Mycobacteria that catalyze aflatoxin degradation have been identified [16]. *Bacillus megaterium* is a rod-shaped, Gram-positive, endospore-forming, aerotolerant species of bacteria. Some researches showed that *B. megaterium* could inhibit the growth of *A. flavus* and the biosynthesis of aflatoxin B_1_ [17]. The effectiveness of *Rhodococcus erythropolis *on AFB_1_ degradation was first reported in 2006 [18]. 

Response surface methodology (RSM), an experimental strategy for seeking the optimum conditions for a multivariable system, described first by Box and Wilson, is a much more efficient technique for optimization [19]. RSM consists of a group of mathematical and statistical procedures that can be used to study relationships between one or more responses and a number of independent variables. RSM defines the effect of the independent variables, alone or in combination, on the process. This method has been successfully applied to the optimization of medium composition, conditions of enzymatic hydrolysis, and parameters of food preservation and fermentation processes.

The purpose of this study was to screen and to establish the optimum conditions for aflatoxin degradation involving the variable factors: temperature, pH, liquid volume, inoculum size, agitation speed, incubation time and to investigate the application of response surface methodology using central composite design to model the degradation of AFB_1_.

## 2. Materials and Methods

### 2.1. Microorgamism and Culture Medium

The bacterial strain (*R. erythropolis* 4.1491) used in this study was obtained from China General Microbiological Culture Collection Center (CGMCC), *R. erythropolis* was cultivated in flasks containing ATYP medium for 58.2 h at 15.3 °C with shaking at 180 rpm. The concentration of the viable organisms was estimated to be 10^8^ colony forming units (CFU)/mL by plate count method.

The ingredients of the ATYP medium for propagating *R. erythropolis* consist of (g/L): KH_2_PO_4_, 1; CaCl_2_, 0.1; NaHCO_3_, 3; CH_3_COONa, 1; MgCl_2_, 0.5; NH_4_Cl, 1; NaCl, 1; C_4_H_4_Na_2_O_4_, 0.5; yeast extract, 0.5; peptone, 0.5. One milliliter trace element solution and 1 mL vitamin solution were supplemented to each liter of the ATYP medium to maintain the necessary nutrient requirement of the cells. The trace element solution consists of (g/L): FeCl_2_·4H_2_O, 1.8; CoCl_2_·6H_2_O, 0.25; NiCl_2_·6H_2_O, 0.01; CuCl_2_·2H_2_O, 0.01; MnCl_2_·4H_2_O, 0.7; ZnCl_2_, 0.1; H_3_BO_3_, 0.5; Na_2_MoO_4_·2H_2_O, 0.03; Na_2_SeO_3_·5H_2_O, 0.01. The ingredients of the vitamin solution consist of (g/L): V_H_, 0.1; nicotinic acid, 0.35; V_B1_, 0.3; *p*-aminobenzoic acid, 0.2; C_8_H_12_N_2_O_2_·2HCl, 0.1; calcium pantothenate, 0.1; V_B12_, 0.05. The pH of the medium was adjusted to 5.56.

The aflatoxigenic strain, *A. flavus* 2810, was kindly provided by Prof. Weijian Zhuang, Fujian Agriculture and Forestry University, China and was maintained on potato dextrose agar (PDA) medium (containing the extract from 200 g boiled potato, 20 g glucose and 20 g agar in 1 liter distilled water) at 4 °C. Spore suspensions were prepared by harvesting seven-day-old sporulating *A. flavus *cultures with sterile distilled water. Spores were counted with a hemocytometer and then were diluted with sterile distilled water to the desired concentration.

### 2.2. Degradation of AFB_1_ by *R. erythropolis* in Liquid Culture

*R. erythropolis* (10^8^ CFU/mL) and 0.5 mL aflatoxin B_1_ (10 mg/L, Sigma, Saint Louis, Missouri, USA) were added to autoclaved ATYP medium in 100 mL flasks. ATYP medium supplemented with AFB_1_ without *R. erythropolis* was used as control. After incubation, the cells were removed by centrifugation at 12000 rpm for 10 min, and AFB_1 _was extracted from the supernatants by methyl alcohol and quantified by aflatoxin B_1_ ELISA test kit (Beacon Analytical Systems Inc., Portland, Maine, USA).

### 2.3. Experimental Design

#### 2.3.1. Plackett-Burman Design

The Plackett–Burman (PB) experimental design is widely used as a screening tool [20]. Compared with the conventional full factorial design, which is labor-intensive and time-consuming, the PB design significantly decreases the number of experiments needed to effectively achieve experimental objectives [21]. The technique is based on the first-order polynomial model:

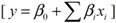
(1)


where y is the response (the degradation rate of AFB_1_), β_0_ is the model intercept and β_i_ is the linear coefficient and x_i_ is the level of the independent variable [22]. The range and the levels of the variables with both coded values and natural values investigated in this study are given in Table 1. The calculation software SAS 8.0 (SAS Inst. Inc, Cary, N.C., USA) was used for the regression analysis of the data obtained.

**Table 1:** Range of values for Plackett-Burman (PB) ^a^.

Code	Variables (unit)		Levels ^a^	
		−1	0	+1
X_1_	Temperature (°C)	15	25	35
X_2_	pH	6.0	7.0	8.0
X_3_	Liquid volume (mL/100-mL)	10	20	30
X_4_	Inoculum size (%)	6	10	14
X_5_	Agitation speed (rpm)	160	180	200
X_6_	Incubation time (h)	48	72	96

^a^ x_1_ = (X_1_ − 25)/10; x_2_ = (X_2_ − 7.0)/1; x_3_ = (X_3_ − 20)/10; x_4_ = (X_4_ − 10)/4; x_5_ = (X_5_ − 180)/20; x_6_ = (X_6_ − 72)/24.

#### 2.3.2. Central Composite Design (CCD) and Response Surface Analysis (RSM)

A Central composite design (CCD) with five coded levels was used for exploring the sub-region of the response surface in the neighborhood of the optimum. The experimental results of the response surface analysis (RSM) were fitted via the response surface exploring the sub-region of the response surface in the neighborhood of the optimum. The experimental results of RSM were fitted via the response surface regression procedure, as expressed by the following second order polynomial equation:

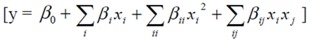
(2)
where y is the predicted response (the degradation rate of AFB_1_), x_i_x_j_ are independent variables, β_0 _is the offset term, β_i_ is the linear coefficient, β_ii_ is the quadratic coefficient, and β_ij_ is the second-order interaction coefficient [23]. Data were analyzed using the response surface regression procedure (SAS 8.0) where x is the coded level of the independent variable.

### 2.4. Inhibition of AFB_1_ by *R. erythropolis* in Peanuts

Peanuts that reach commercial level of maturity were harvested and used immediately or stored at 4 °C for use within 48 h. For preparation, the peanut kernels were washed with tap water, then surface-disinfected with 0.1% sodium hypochlorite for 1 min, cleaned with tap water and air dried [17]. Twenty grams of peanuts were placed in each of the four autoclaved 100 mL flasks (three treatments plus one control). The three treatments were: (A) Two milliliters of *R. erythropolis* cell suspension (10^8^ CFU/mL); (B) 2 mL *B. megaterium* cell suspension (10^8^ CFU/mL); (C) 1 mL each of *R. erythropolis* and *B. megaterium* cell suspension (10^8^ CFU/mL); and (D) sterile distilled water (control). After incubation at room temperature for three hours, 200 μL of *A. flavus* spores at a concentration of 10^6^ spores/mL were inoculated into each flask. The flasks were placed in growth chamber (QHX-300BS-III, Shanghai Xinmiao) with controlled humidity at 85% and temperature at 30 °C for incubation. The concentration of aflatoxin B_1_ was examined after seven days’ inoculation using high performance liquid chromatography (HPLC) [24]. 

## 3. Results and Discussion

### 3.1. Plackett-Burman Design

The design matrix selected for the screening of significant variables for the degradation of AFB_1_ and the corresponding responses are shown in Table 2. The adequacy of the model was calculated, and the variables evidencing statistically significant effects were screened via Student’s *t*-test for ANOVA (Table 3). It is indicated that temperature, pH, liquid volume and incubation time were the most important factors. The polynomial model describing the correlation between the formulation and the variables (x_1_ − x_6_) and the degradation rate of AFB_1_ (y) can be expressed by the following equation:

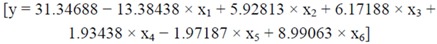
(3)


**Table 2:** Experimental designs and the results of the PB design.

Run	x_1_	x_2_	x_3_	x_4_	x_5_	x_6_	y (%)
1	−1	−1	−1	−1	−1	−1	18.1
2	1	−1	−1	−1	−1	1	10.6
3	−1	1	−1	−1	−1	1	80.9
4	1	1	−1	−1	−1	−1	12.6
5	−1	−1	1	−1	−1	1	75.0
6	1	−1	1	−1	−1	−1	13.4
7	−1	1	1	−1	−1	−1	26.2
8	1	1	1	−1	−1	1	11.8
9	−1	−1	−1	1	−1	1	51.4
10	1	−1	−1	1	−1	−1	10.9
11	−1	1	−1	1	−1	−1	27.2
12	1	1	−1	1	−1	1	11.3
13	−1	−1	1	1	−1	−1	49.0
14	1	−1	1	1	−1	1	9.2
15	−1	1	1	1	−1	1	90.6
16	1	1	1	1	−1	−1	34.9
17	−1	−1	−1	−1	1	1	26.6
18	1	−1	−1	−1	1	−1	15.8
19	−1	1	−1	−1	1	−1	14.5
20	1	1	−1	−1	1	1	12.5
21	−1	−1	1	−1	1	−1	22.9
22	1	−1	1	−1	1	1	22.8
23	−1	1	1	−1	1	1	85.7
24	1	1	1	−1	1	−1	21.2
25	−1	−1	−1	1	1	−1	12.2
26	1	−1	−1	1	1	1	8.7
27	−1	1	−1	1	1	1	62.8
28	1	1	−1	1	1	−1	26.7
29	−1	−1	1	1	1	1	40.3
30	1	−1	1	1	1	−1	19.8
31	−1	1	1	1	1	−1	32.3
32	1	1	1	1	1	1	45.2
33	0	0	0	0	0	0	79.4
34	0	0	0	0	0	0	81.4
35	0	0	0	0	0	0	79.8
36	0	0	0	0	0	0	80.9

**Table 3:** Identifying significant variables for the degradation of AFB_1_ using Plackett–Burman design.

Variable	Coefficients	*t* Value	*p*-value
Intercept	31.34688	10.52	<0.0001
x_1_	−13.38438	−4.49	0.0001
x_2_	5.92813	1.99	0.0578
x_3_	6.17188	2.07	0.0489
x_4_	1.93438	0.65	0.5223
x_5_	−1.97187	−0.66	0.5143
x_6_	8.99063	3.02	0.0058

The results of the *t*-test for variance between the average of observation of the 2-level experiment and the center point showed that the difference was significant (*P* < 0.05). This indicated that the optimum point was in the domain of our experiment. The next step in the optimization of the degradation efficiencies was to determine the optimum levels of significant variables. For this purpose, the RSM, using a central composite design, was adopted for optimization of the degradation efficiencies.

### 3.2. Central Composite Design and Response Surface Analysis

A central composite design (CCD) under RSM was used to analyze the interactive effect of temperature, pH, liquid volume and incubation time to reach an optimum level. The design matrix and the corresponding results of the RSM experiments to determine the effects of four independent variables are shown in Table 4. 

**Table 4:** The matrix of the central composite design (CCD) experiment and the corresponding experimental data.

Runs	x_1_	x_2_	x_3_	x_6_	y (%)
1	−1	−1	−1	−1	57.6
2	−1	−1	−1	1	47.8
3	−1	−1	1	−1	74.9
4	−1	−1	1	1	80.4
5	−1	1	−1	−1	64.2
6	−1	1	−1	1	72.4
7	−1	1	1	−1	65.3
8	−1	1	1	1	90.4
9	1	−1	−1	−1	47.0
10	1	−1	−1	1	46.3
11	1	−1	1	−1	64.8
12	1	−1	1	1	62.0
13	1	1	−1	−1	54.6
14	1	1	−1	1	59.7
15	1	1	1	−1	60.5
16	1	1	1	1	65.1
17	−2	0	0	0	68.1
18	2	0	0	0	53.7
19	0	−2	0	0	69.1
20	0	2	0	0	92.2
21	0	0	−2	0	68.5
22	0	0	2	0	95.5
23	0	0	0	−2	60.7
24	0	0	0	2	95.8
25	0	0	0	0	82.6
26	0	0	0	0	84.4
27	0	0	0	0	83.7
28	0	0	0	0	83.9
29	0	0	0	0	83.4
30	0	0	0	0	82.2
31	0	0	0	0	84.0

x_1_ = (X_1_ − 25)/5; x_2_ = (X_2_ − 7.0)/0.5; x_3_ = (X_3_ − 20)/5; x_6_ = (X_6_ − 72)/12.

Experimental data were used in the response surface regression (RSREG) procedure of SAS to find the coefficients of the response function. The coefficients of the response function for the degradation efficiencies are listed in Table 5. In this case, temperature, pH, liquid volume and incubation time had a significant effect on degradation of AFB_1_ (*P* < 0.05), as well as the quadratic terms of temperature. The fitness of the model was examined by the coefficient of determination *R^2^*, which was found to be 0.8096, indicating that the sample variation of 80.96% was attributed to the variables. The model can be shown as follows:

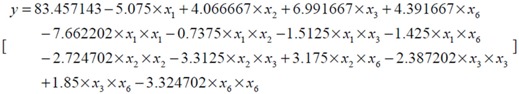
(4)


Table 6 shows the analysis of variance of the regression parameters of the predicted response surface quadratic models for the degradation of AFB_1_. As can be seen, both linear and quadratic parameters were very significant. However, the statistical analysis showed that the interaction among parameters was insignificant. This means that all factors considered in this study had a specific effect on the degradation. However, there were no effects of multi-variable interaction present. The effect of individual variable factors is more significant than that of the multi-factor interaction [25].

**Table 5:** Regression coefficients of the response function for the degradation of AFB_1_.

Parameter	DF	Estimate	StandardError	*t* Value	Pr > |*t*|
Intercept	1	83.457143	3.273286	25.50	<0.0001
x_1_	1	−5.075000	1.767777	−2.87	0.0111
x_2_	1	4.066667	1.767777	2.30	0.0352
x_3_	1	6.991667	1.767777	3.96	0.0011
x_6_	1	4.391667	1.767777	2.48	0.0244
x_1_ * x_1_	1	−7.662202	1.619505	−4.73	0.0002
x_2_ * x_1_	1	−0.737500	2.165075	−0.34	0.7378
x_2_ * x_2_	1	−2.724702	1.619505	−1.68	0.1119
x_3_ * x_1_	1	−1.512500	2.165075	−0.70	0.4948
x_3 _* x_2_	1	−3.312500	2.165075	−1.53	0.1456
x_3_ * x_3_	1	−2.387202	1.619505	−1.47	0.1599
x_6_ * x_1_	1	−1.425000	2.165075	−0.66	0.5198
x_6_ * x_2_	1	3.175000	2.165075	1.47	0.1619
x_6_ * x_3_	1	1.850000	2.165075	0.85	0.4055
x_6_ * x_6_	1	−3.324702	1.619505	−2.05	0.0568

**Table 6:** ANOVA results for central composite design (CCD).

Regression	DF	Type I Sum of Squares	*R*-Square	*F* Value	Pr > *F*
Linear	4	2651.125000	0.4205	8.84	0.0006
Quadratic	4	1983.513233	0.3146	6.61	0.0024
Crossproduct	6	469.407500	0.0745	1.04	0.4345
Total Model	14	5104.045733	0.8096	4.86	0.0017

**Figure 1:**
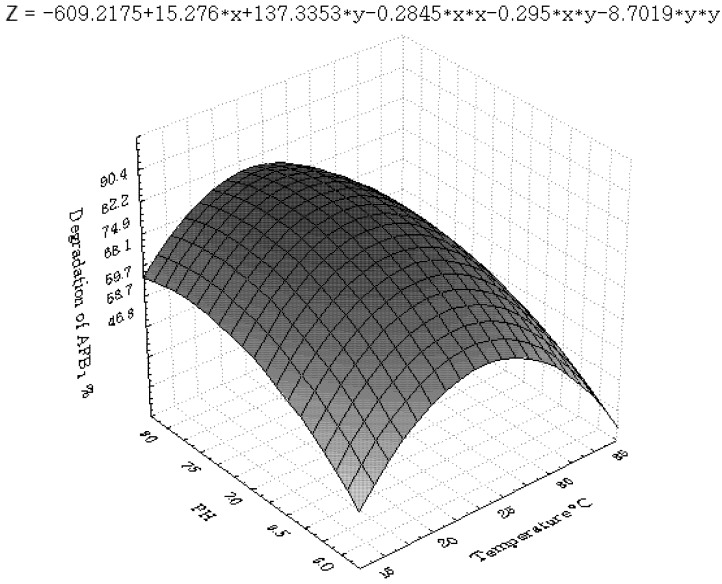
The response surface plot showing the effects of temperature (x_1_) and pH (x_2_) on AFB_1_ degradation.

**Figure 2:**
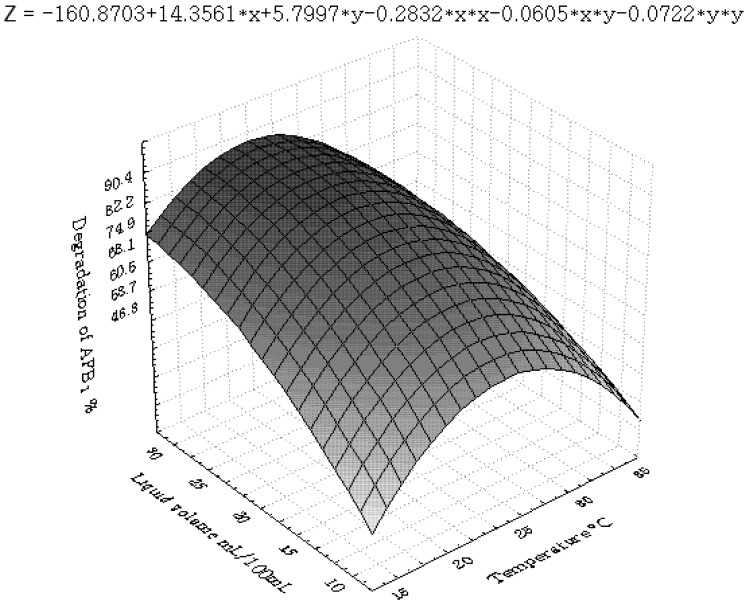
The response surface plot showing the effects of temperature (x_1_) and liquid volume (x_3_) on AFB_1_ degradation.

**Figure 3:**
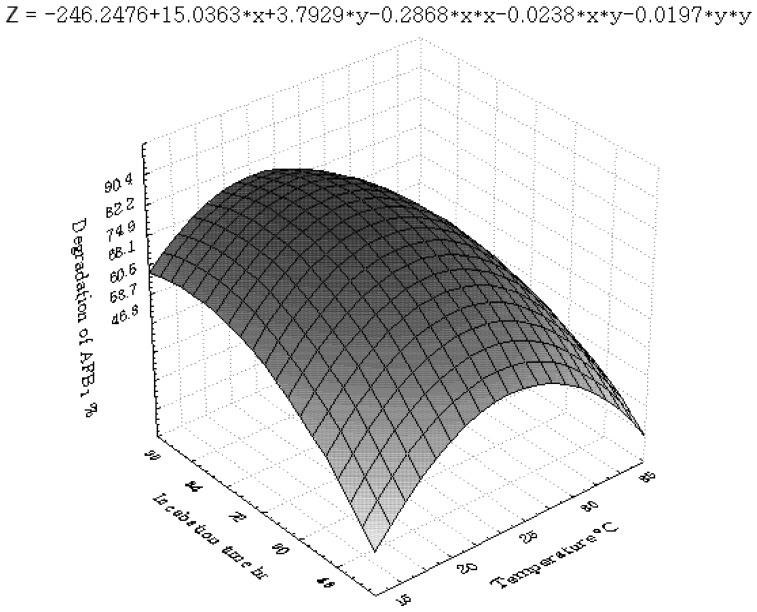
The response surface plot showing the effects of temperature (x_1_) and incubation time (x_6_) on AFB_1_ degradation.

**Figure 4:**
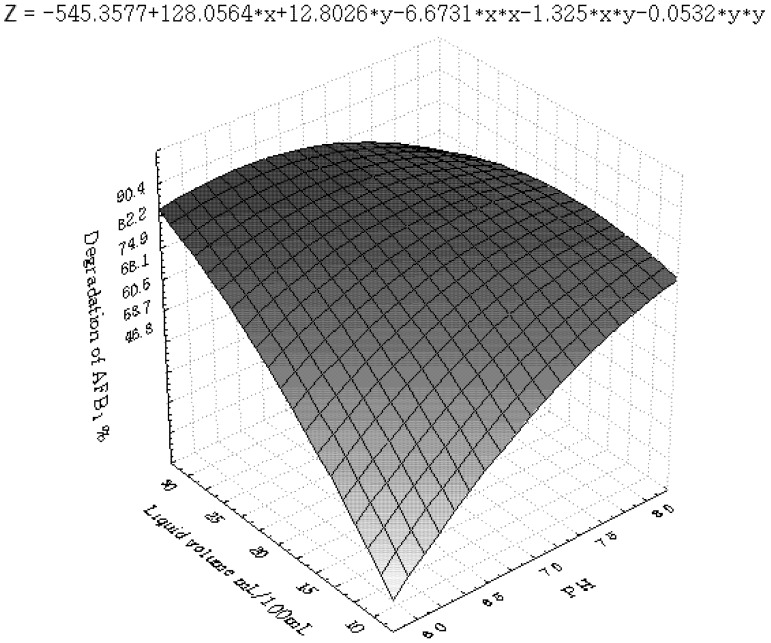
The response surface plot showing the effects of pH (x_2_) and liquid volume (x_3_) on AFB_1_ degradation.

**Figure 5:**
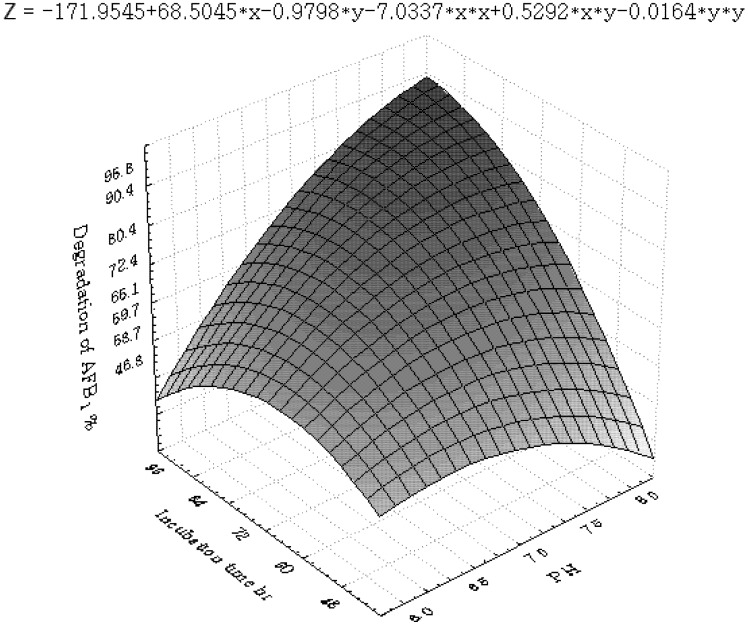
The response surface plot showing the effects of pH (x_2_) and incubation time (x_6_) on AFB_1_ degradation.

**Figure 6:**
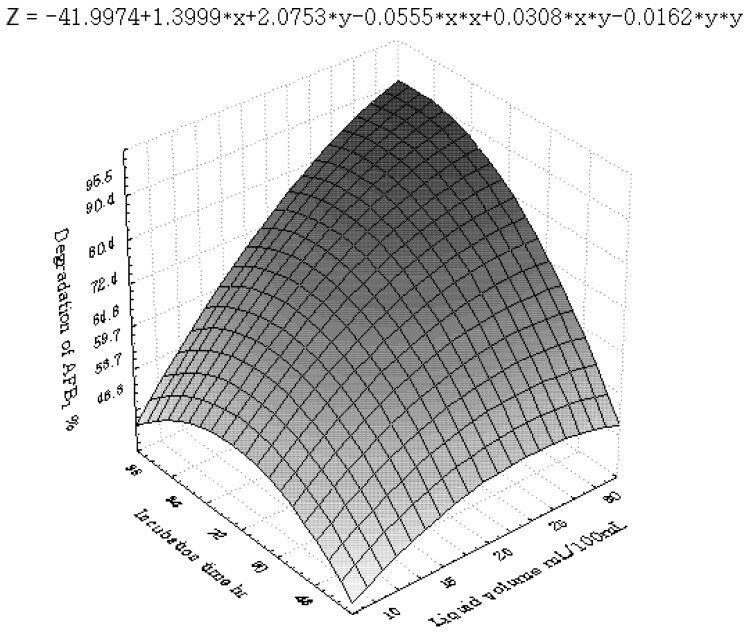
The response surface plot showing the effects of liquid volume (x_3_) and incubation time (x_6_) on AFB_1_ degradation.

Figure 1, Figure 2, Figure 3, Figure 4, Figure 5 and Figure 6 portray the 3D response surface plots constructed using the quadratic regression model generated by regression analysis. Figure 1 shows the effects of temperature and pH on the AFB_1_degradation_. _The degradation of AFB_1_ increased with temperatures up to 25 °C. In temperature ranges over 25 °C, a negative effect of the temperature on AFB_1 _degradation was observed. AFB_1 _degradation increased with an increase in pH. The effects of temperature and liquid volume are shown in Figure 2. An increase in temperature above 25 °C resulted in less degradation of AFB_1_. As the liquid volume increased, the degradation of AFB_1_ increased at all temperatures probably due to the fact that a high liquid volume is conducive to the growth of bacteria. Figure 3 shows the effects of the interaction of temperature and incubation time on the degradation of AFB_1_. As the length of the incubation time increased to 84 h, the degradation of AFB_1_ increased at all temperatures. However, further increase in incubation time resulted in little increase in the degradation of AFB_1_. Figure 4 is a response surface plot indicating the effects of pH and liquid volume on the degradation of AFB_1_. pH and liquid volume had a positive linear effect on the degradation of AFB_1_. The plot also reveals that properly increasing pH is conducive to increasing degradation. Figure 5 shows the effects of the interaction of pH and incubation time on the degradation of AFB_1_. At 96 h, a linear increase in degradation was observed when the pH was increased. In alkaline conditions, increase in incubation time resulted in increased degradation. A similar profile is observed in Figure 6 with liquid volume and incubation time.

According to the canonical analysis, the results predicted by the model showed that the maximum degradation could be achieved when the temperature, initial pH, liquid volume and incubation time were set at 23.2 °C, 7.17, 24.6 mL/100 mL and 81.9 h, respectively. The predicted optimum rate of AFB_1_ degradation was 96.7%. In order to confirm the optimization results, a further degradation test was carried out under the optimal conditions based on the results from the model; the experimentally observed optimum rate of AFB_1_ degradation was 95.8% which was quite in agreement with the predicted value.

### *3.3. Inhibition of AFB_1_ by *R. erythropolis *in Peanuts*

The strain of marine *B. megaterium* was isolated from the Yellow Sea of East China, and showed reduced postharvest infection of peanut kernels caused by *A. flavus* [17]. The results showed both *R. erythropolis* and *B. megaterium* could significantly reduce the biosynthesis of aflatoxins in peanuts (*p* < 0.01) (Table 7). The effect of *R. erythropolis* plus *B. megaterium* was better than that of a single bacterial strain. 

**Table 7:** Inhibition of aflatoxin B_1_ by *R. erythropolis* in peanuts.

	Control	*R. erythropolis*	*B. megaterium*	*R. erythropolis* and *B. megaterium*
Aflatoxin B_1_ (μg/kg) (mean ± SD)	195.69 ± 1.92	178.38 ± 2.47	148.27 ± 3.87	140.80 ± 3.59

Tejada-Castañeda *et al.*, reported that a strain of *Nocardia corynebacteroides* was safe to detoxify aflatoxin-contaminated feed for chickens, because it could transform AFB_1_ to other less toxic compounds [26]. Can *R. erythropolis* degrade AFB_1_ completely or can it only transform AFB_1_ to other less toxic compounds? To answer this question, the metabolites of AFB_1_ incubated with *R. erythropolis *should be studied further. Furthermore, the work of Taylor *et al.*, describes the enzymes in *Mycobacterium smegmatis* that degrade aflatoxin, which is subsequently corroborated by the work of Lapalikar *et al.*, who show that enzymes from *R. erythropolis* also degrade aflatoxin [17,27]. These enzymes are dependent upon the cofactor F_420_, whose production has been shown to be maximal at around 96 hours [28,29]. In a next step, the enzyme activity of F_420_H_2_-dependent reductases in the strain of *R. erythropolis* used in this study and the expression of aflatoxin pathway genes in *A. flavus* corresponding to the effect of *R. erythropolis* should be studied in greater detail, because the results could help us elucidate the mechanism of degradation of aflatoxin by *R. erythropolis*.

## 4. Conclusions

Plackett–Burman design and central composite design were adopted to screen the key factors and identify the optimal conditions for degradation of AFB_1_. Using RSM analysis, the four significant variables (temperature, pH, liquid volume and incubation time) selected by the Plackett–Burman design experiment were found to have linear effects on AFB_1_ degradation at significant level. The optimum conditions of each variable were as follows: temperature, 23.2 °C; pH, 7.17; liquid volume, 24.6 mL/100mL; inoculum size, 10%; agitation speed, 180 rpm; and incubation time, 81.9 h. Under these conditions, the AFB_1_ degradation efficiency of *R. erythropolis* was increased from 28.7% to 95.8%.

Our result by mathematic modeling has great potential for practical applications. It can be used in aflatoxin removal during industrial fermentation for food processing and fermentation for bioenergy generation such as ethanol production. *R. erythropolis* can be used to reduce the biosynthesis of aflatoxins in peanuts and other agricultural food commodities. It can potentially be used in biocontrol to reduce aflatoxin contamination of food and feed utilizing its activity against the aflatoxins biosynthesis in *A. flavus*. The potential for commercial use in the market to prolong shelf life can also be explored.
